# Disulfidptosis-related signature elucidates the prognostic, immunologic, and therapeutic characteristics in ovarian cancer

**DOI:** 10.3389/fgene.2024.1378907

**Published:** 2024-04-17

**Authors:** Yunyan Cong, Guangyao Cai, Chengcheng Ding, Han Zhang, Jieping Chen, Shiwei Luo, Jihong Liu

**Affiliations:** ^1^ Department of Oncology, The Fifth Affiliated Hospital of Sun Yat-Sen University, Zhuhai, China; ^2^ Department of Gynecologic Oncology, State Key Laboratory of Oncology in South China, Guangdong Provincial Clinical Research Center for Cancer, Sun Yat-Sen University Cancer Center, Guangzhou, China; ^3^ Guangdong Provincial Clinical Research Center for Obstetrical and Gynecological Diseases, Guangzhou, China; ^4^ Department of Obstetrics and Gynecology, The First Affiliated Hospital of Sun Yat-Sen University, Guangzhou, China

**Keywords:** disulfidptosis, ovarian cancer, prognosis, treatment, biomarker

## Abstract

**Introduction:**

Ovarian cancer (OC) is the deadliest malignancy in gynecology, but the mechanism of its initiation and progression is poorly elucidated. Disulfidptosis is a novel discovered type of regulatory cell death. This study aimed to develop a novel disulfidptosis-related prognostic signature (DRPS) for OC and explore the effects and potential treatment by disulfidptosis-related risk stratification.

**Methods:**

The disulfidptosis-related genes were first analyzed in bulk RNA-Seq and a prognostic nomogram was developed and validated by LASSO algorithm and multivariate cox regression. Then we systematically assessed the clinicopathological and mutational characteristics, pathway enrichment analysis, immune cell infiltration, single-cell-level expression, and drug sensitivity according to DRPS.

**Results:**

The DRPS was established with 6 genes (MYL6, PDLIM1, ACTN4, FLNB, SLC7A11, and CD2AP) and the corresponding prognostic nomogram was constructed based on the DRPS, FIGO stage, grade, and residual disease. Stratified by the risk score derived from DRPS, patients in high-risk group tended to have worse prognosis, lower level of disulfidptosis, activated oncogenic pathways, inhibitory tumor immune microenvironment, and higher sensitivity to specific drugs including epirubicin, stauroporine, navitoclax, and tamoxifen. Single-cell transcriptomic analysis revealed the expression level of genes in the DRPS significantly varied in different cell types between tumor and normal tissues. The protein-level expression of genes in the DRPS was validated by the immunohistochemical staining analysis.

**Conclusion:**

In this study, the DRPS and corresponding prognostic nomogram for OC were developed, which was important for OC prognostic assessment, tumor microenvironment modification, drug sensitivity prediction, and exploration of potential mechanisms in tumor development.

## 1 Introduction

Ovarian cancer (OC) is the fifth leading cause of cancer death in women worldwide and represents a significant public health challenge (Siegel et al., 2023). Although most OCs were sensitive to platinum-based chemotherapy, about 80% cases developed resistance with a poor survival outcome (Pignata et al., 2019). Therefore, it is crucial to identify biomarkers for prognosis prediction and individualized treatment-guidance and reveal the mechanism underlying the development and metastasis in OC.

Cell death is a natural process that occurs in various physiological and pathological conditions, which has been proved to play a role in the elimination of damaged or cancerous cells, and regulate anti-cancer immunity in the tumor microenvironment (TME) (Peng et al., 2022). Over ten different types of cell death have been reported, which can be thoroughly classified as the regulatory cell death (RCD) and accidental cell death (ACD). Disulfidptosis is a newly discovered type of RCD, which is characterized by the actin cytoskeleton collapse caused by disulfide stress (Liu et al., 2023; Machesky, 2023). Under glucose starvation, NADPH depletion could induce excessive accumulation of intracellular disulfide molecules, leading to aberrant disulfide-bond formation in actin cytoskeleton and F-actin contraction, which triggered potent cell death. Some cancer cells might be more susceptible to disulfidptosis compared with normal tissue for its upregulated SLC7A11 (Li et al., 2022) and NADPH-dependent metabolism (Ju et al., 2020). In the context of OC, studying disulfidptosis would provide insights into its involvement in tumor initiation, progression, metastasis, and the interactions between cancer cells and the immune system.

In this study, we would like to develop a disulfidptosis-related prognostic signature (DRPS) for OC, explore the effects of disulfidptosis-related risk stratification, and discuss the potential therapeutic target for patients in different risk groups.

## 2 Materials and methods

### 2.1 Data sources

The gene expression data in counts format and corresponding clinical data of TCGA was downloaded from UCSC Xena (https://xenabrowser.net/datapages/?dataset= TCGA-OV.htseq_counts.tsv&host=https%3A%2F%2Fgdc.xenahubs.net&removeHub=https%3A%2F%2Fxena.treehouse.gi.ucsc.edu%3A443), which was then transformed from the raw counts to the count-per- million (cpm) value for later DRPS construction. The combined data of TCGA and GTEx in DESeq2 normalized counts format was downloaded from UCSC Xena (https://xenabrowser.net/datapages/?dataset=TCGA-GTEx-TARGET-gene-exp-counts.deseq2-normalized.log2&host=https%3A%2F%2Ftoil.xenahubs.net&removeHub=https%3A%2F%2Fxena.treehouse.gi.ucsc.edu%3A443) The somatic mutation data of TCGA-OV was downloaded from GDC Data Portal (https://portal.gdc.cancer.gov/). The GSE9891 and E-MTAB-386 datasets used for validation were downloaded from https://www.ncbi.nlm.nih.gov/geo/ and https://www.ebi.ac.uk/biostudies/, separately. Totally, we included 374, 285, and 129 patients with OC from TCGA database, GSE9891, and E-MTAB-386 datasets.

### 2.2 Disulfidptosis-related prognostic model construction and validation

The eighteen disulfidptosis-related genes were obtained from Liu et al. (2023), including four suppressor hits (SLC7A11, SLC3A2, RPN1, and NCKAP1) and fourteen core actin-related proteins (INF2, CD2AP, PDLIM1, ACTN4, MYH9, MYH10, IQGAP1, FLNA, FLNB, TLN1, MYL6, ACTB, DSTN, and CAPZB). The disulfidptosis-related prognostic model for OC was developed by two steps in the TCGA-OV cohort: First, we conducted least absolute shrinkage and selection operator (LASSO) method with the “glmnet” package (version 4.1–7) to select the robust prognosticators among 18 candidate genes using cpm format data (transformed from the raw counts) (Friedman et al., 2010). The best lambda value in the LASSO regularization was chosen by 10-fold cross-validation. Second, Cox model with forward stepwise regression was performed to build the DRPS and the corresponding prognostic model with “survival” package (version 3.5–5) (Therneau and Grambsch, 2000). Patients were stratified into high- and low-risk groups based on whether their risk scores were higher than the median of the whole cohort (high risk and worse prognosis) or less than the median of the whole cohort (low risk and better prognosis). The model was subsequently validated in two independent external datasets: GSE9891 and E-MTAB-386. The significance of the prognosis between these two groups was evaluated through the application of Kaplan-Meier survival analysis and the *p*-value was calculated using the log-rank test. In order to enhance the accuracy of prediction, we incorporated the FIGO stage, grade, and residual disease, along with the disulfidptosis-related risk score, to construct a nomogram using “survival” package (version 3.5–5) and “regplot” package (version 1.1) (Therneau and Grambsch, 2000). The efficacy of this nomogram was subsequently validated through the Receiver-operator characteristic (ROC) curve using “timeROC” package (version 0.4) (Blanche et al., 2013).

### 2.3 Mutation, phenotype, and disulfidptosis characteristics

Mutation signature of high- and low-risk groups was analyzed by the R package “maftools” (Mayakonda et al., 2018). Six phenotype characteristics were included in the comparison, including survival status, FIGO stage, histological grade, tumor residual size, homologous recombination deficiency (HRD) status, and germline BRCA mutation status (gBRCAmut). Since SLC7A11, SLC3A2, RPN1, and NCKAP1 had been verified as suppressor hits of disulfidptosis, we treated these four genes as a geneset and calculated the gene set variation analysis (GSVA) scores per patient for it to show the disulfidptosis level (Hänzelmann et al., 2013).

### 2.4 Differential gene expression and functional pathway enrichment analysis

Differential gene expression analysis between high- and low-risk groups stratified by DRPS followed the limma-voom pipeline using raw counts data (Ritchie et al., 2015). Significant differentially expressed genes (DEGs) were defined as genes whose FDR were less than 0.05 and absolute value of log2 fold change greater than 1. Enrichment analysis of Kyoto Encyclopedia of Genes and Genomes (KEGG) and Gene Ontology (GO) pathways were conducted separately on the significant DEGs between risk groups stratified by DRPS and genes included in the DRPS to identify functional pathways by the “clusterProfiler” package (version 4.8.1) (Wu et al., 2021). Gene set enrichment analysis (GSEA) in HALLMARK gene pathways was also performed with the “clusterprofiler” package. Annotated clusters were ranked according to Group Enrichment Score and *p*-value. Pearson correlation coefficients between genes in DRPS were calculated by “Hmisc” package (version 5.1-0). Sensitivity analysis of the DRPS was performed using copula-based methods (Yeh et al., 2023).

We calculated the DEGs between OC and normal ovary using limma method. OC samples and normal ovary samples were selected from the combined TCGA and GTEx expression matrix in DESeq2 normalized counts format for further comparison. DEGs between OC and normal ovary were identified as absolute value of log2 fold change >2 and FDR <0.001 from the result of limma.

### 2.5 Tumor immune microenvironment analysis and immunotherapy prediction

The immune cell infiltration in TME was evaluated by TIMER 2.0 (http://timer.cistrome.org/) (Li et al., 2017), CIBERSORT (Chen et al., 2018), xCell (Aran et al., 2017), EPIC (Racle and Gfeller, 2020), quanTIseq (Finotello et al., 2019), MCPcounter (Becht et al., 2016). Since there’s no available OC cohort with immunotherapy response, the published markers as well as TIDE score were used to predict the potential immune checkpoint inhibitor (ICI) response (Jiang et al., 2018).

### 2.6 Single-cell landscape of disulfidptosis-related risk gene expression and identification of cell types associated with disulfidptosis-related risk stratification

Single-cell RNA sequencing data was obtained from EMBL-EBI (https://www.ebi.ac.uk/biostudies/arrayexpress) (Qian et al., 2020), including 44532 cells from 5 patients with primary OC (7 OC samples and 3 normal samples). Data quality control was conducted with the primary filtering condition of nFeature_RNA >200 & nFeature_RNA <6000 & percent. mt < 25 & percent. HB < 10 and DoubletFinder (version 2.0.3). Clustering was performed using the “Seurat” package (version 4.3.0.1) at a revolution of 0.5 (Butler et al., 2018). According to published studies, we used a set of marker genes to determine cell types: EPCAM, KRT7, KRT8, KRT17, SPRR3 for epithelial/tumor cells; CD3E, CD3D, TRBC1/2, TRAC for T cells; LYZ, CD86, CD68, FCGR3A for myeloid cells; CD79A/B, JCHAIN, IGKC, IGHG3 for B cells and plasma cells; CLDN5, FLT1, CDH1, RAMP2 for endothelial cells; DCN, C1R, COL1A1, ACTA2 for fibroblasts; TAGLN, CNN1 for smooth muscle cells (Dinh et al., 2021).

### 2.7 Drug sensitivity and prediction

The drug sensitivity was predicted by half-maximal inhibitory concentration (IC50) according to GDSC data (https://www.cancerrxgene.org/) using the “oncoPredict” package (version 0.2) (Maeser et al., 2021). The potential targeted drug for risk groups was assessed by Drug-Gene Interaction database (DGIdb) (https://www.dgidb.org/) (Freshour et al., 2021).

### 2.8 Immunohistochemical staining analysis

We downloaded the immunohistochemical (IHC) staining images of OC and normal ovaries from the Human Protein Atlas (HPA) (https://www.proteinatlas.org/). ImageJ (version 1.8.0) was used to analyze the integrated optical density (IOD) and average optical density (AOD).

### 2.9 Statistical analyses

R version 4.2.2 was used for statistical analyses. We utilized WilcFoxon test to evaluate the statistical significance for continuous characteristics, and Chi-square test or Fisher’s exact test for categorical characteristics as appropriate. Two-tail *p*-value <0.05 was set as statistically significant. The R script used in this study was uploaded in Supplementary Materials, R script.

## 3 Results

### 3.1 Disulfidptosis-related prognostic signature and prediction model construction and validation

**FIGURE 1:**
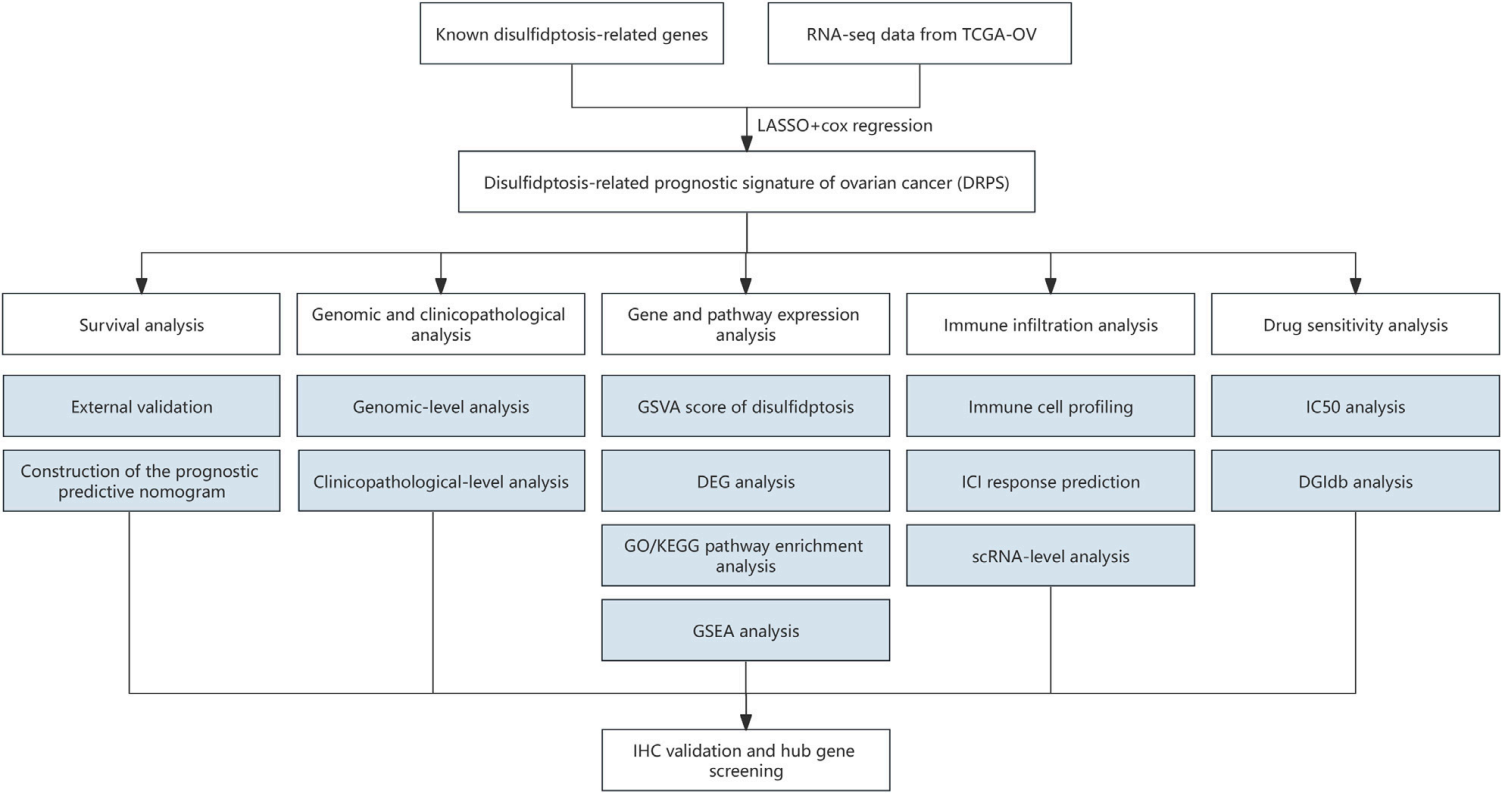
The flowchart of the study. (RNA-seq, RNA sequencing. LASSO, least absolute shrinkage and selection operator. GSVA, the gene set variation analysis. DEG, differentially expressed genes. GO, Gene Ontology. KEGG, Kyoto Encyclopedia of Genes and Genomes. GSEA, Gene set enrichment analysis. IHC, immunohistochemical. ICI, immune checkpoint inhibitor. scRNA, Single-cell RNA. IC50, half-maximal inhibitory concentration. DGIdb, Drug-Gene Interaction database.).

**FIGURE 2:**
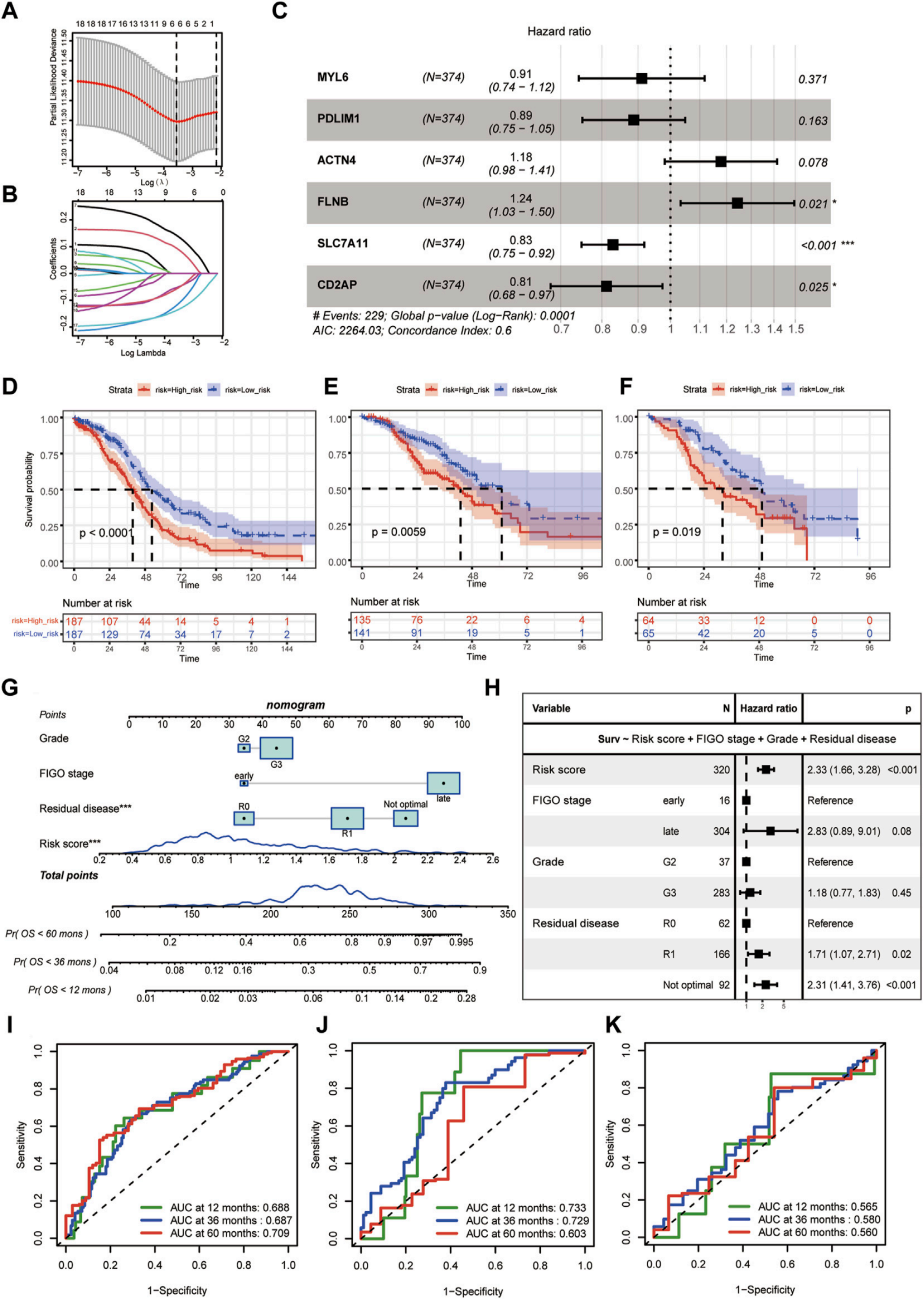
Disulfidptosis-related prognostic model construction. **(A)** Selection of lambda value in the LASSO analysis for OS using ten-fold cross-validation by the minimum criteria. **(B)** LASSO coefficient profiles of candidate genes for OS. **(C)** Hazard ratios of the final included six disulfidptosis-related genes after multivariate cox regression adjustment for OS. **(D–F)** The Kaplan-Meier curves of disulfidptosis-related high- and low-risk groups for OS in TCGA-OV **(D)**, GSE9891 **(E)**, E-MTAB-386 **(F)**. **(G)** The predictive nomogram based on disulfidptosis-related risk score and clinicopathological factors. **(H)** Hazard ratios of the final included variables in the nomogram after multivariate cox regression adjustment for OS. **(I–K)** Receiver-operating curves (ROC curves) for 12-, 36-, and 60- month OS of the predictive nomogram in TCGA-OV **(I)**, GSE9891 **(J)**, E-MTAB-386 **(K)**. (LASSO, least absolute shrinkage and selection operator. OS, overall survival).

The flowchart of this study was shown in Figure 1. The baseline characteristics of the datasets showed that epithelial (serous) was the main histologic type and grade 3 was the main pathologic grade in all the three cohorts, consistent with global real-world reports (Supplementary Table S1) (Kuroki and Guntupalli, 2020). Six genes (MYL6, PDLIM1, ACTN4, FLNB, SLC7A11, and CD2AP) were selected by the LASSO and then used as the input of multivariate Cox regression analysis to build the DRPS (Figures 2A, B). According to Figure 2C, FLNB was an independent risk factor for overall survival (OS), while SLC7A11 and CD2AP were the independent protective factors. Kaplan-Meier curves for OS illustrated that the prognosis differed markedly between the high- and low-risk groups stratified by the DRPS not only in the training set (*p*-value < 0.0001) but also in two validation sets (*p*-value = 0.0059 for GSE9891, *p*-value = 0.019 for E-MTAB-386) (Figures 2D–F).

Besides, we calculated the risk score of the DRPS by multivariate cox regression. The final disulfidptosis-related prognostic model was developed using the risk score, histological grade, FIGO stage, and residual disease, which was then generated as a nomogram (Figure 2G). As Figure 2H showed, the risk score was an independent risk factor for OS prediction after adjusting those clinicopathological factors. ROC curves further demonstrated that the disulfidptosis-related prognostic model achieved satisfactory performance in the training dataset and validation datasets (Figures 2I–K).

### 3.2 The genomic-level and clinicopathological differences between disulfidptosis-related high- and low-risk groups

**FIGURE 3:**
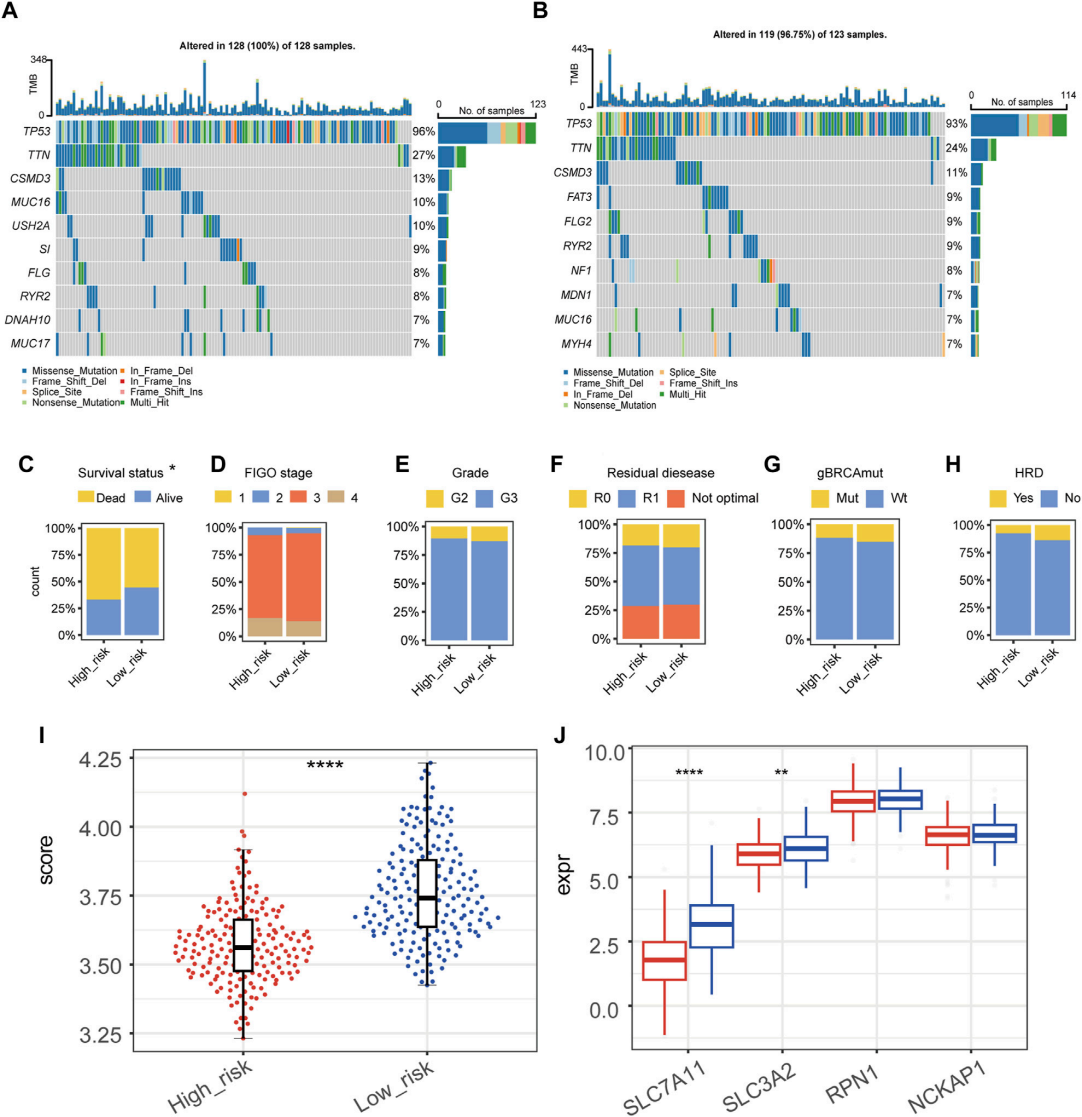
Genomic and clinicopathological characteristics of high- and low-risk groups. **(A,B)** The mutation and copy-number variation combined landscape of high-risk **(A)** and low-risk **(B)** groups of patients with epithelial ovarian cancer. **(C–H)** Distribution of survival status **(C)**, FIGO stage **(D)**, grade **(E)**, residual disease **(F)**, germline BRCA mutation **(G)**, homologous recombination deficiency (HRD) **(H)** in high- and low-risk groups, respectively. **(I)** Disulfidptosis score of high- and low-risk groups by the gene set variation analysis (GSVA). **(J)** RNA expression level of SLC7A11, SLC3A2, RPN1, and NCKAP1 in high- and low-risk groups. (****p-value < 0.0001, **p-value < 0.01).

Top ten genes with the highest frequency in simple nucleotide variation (SNV) and copy number variation (CNV) were displayed in Figure 3A (high-risk group) and Figure 3B (low-risk group). None of these genes were found to have a significant difference between groups, which indicated that the difference in tumor aggressiveness between groups was more associated with the heterogeneity at the transcriptomic-level rather than the genomic-level. For clinicopathological factors, high-risk group had a higher mortality rate compared with low-risk group (Figure 3C); while FIGO stage, histological grade, residual disease, gBRCAmut, and HRD showed no significantly different distribution between groups (Figures 3D–H).

We calculated the GSVA score of disulfidptosis in each sample using the gene set which included SLC7A11, SLC3A2, RPN1, and NCKAP1 (Figure 3I). As the result, high-risk group showed a statistically lower GSVA score of disulfidptosis compared with low-risk group, suggesting a relatively low level of disulfidptosis in high-risk group. Among the four genes used in the GSVA score of disulfidptosis, the expression of SLC7A11 and SLC3A2 were significantly lower in high-risk group (Figure 3J).

### 3.3 Gene and pathway expression characteristics in disulfidptosis-related high- and low-risk groups

**FIGURE 4:**
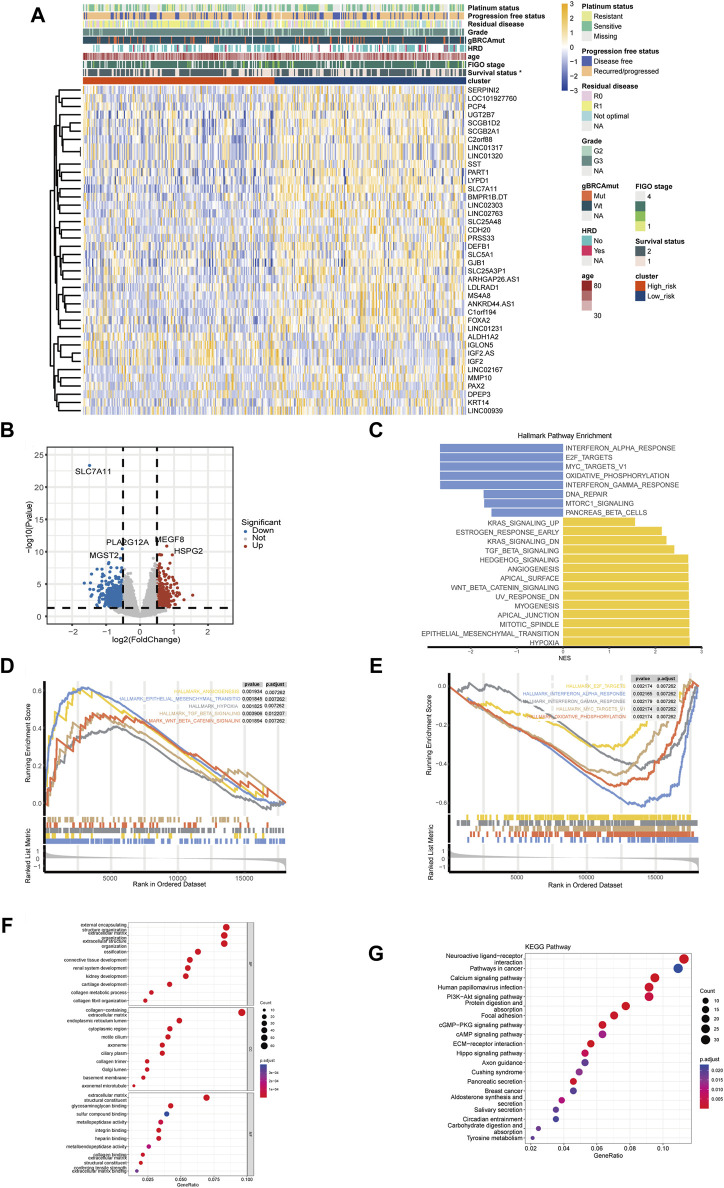
Differentially expressed genes and functional pathway enrichment analysis. **(A)** Heatmap of top differentially expressed genes (DEGs) between high- and low-risk groups. **(B)** Volcano plots of DEGs related to the disulfidptosis-related risk stratification. **(C)** Gene set enrichment analysis (GSEA) in HALLMARK pathways of DEGs between high- and low-risk groups. **(D)** Top 5 enriched HALLMAEK pathways of ovarian cancer patients in high-risk groups. **(E)** Top 5 enriched HALLMAEK pathways of ovarian cancer patients in low-risk group. **(F)** Enrichment analysis of Gene Ontology (GO) pathways between high- and low-risk groups. **(G)** Enrichment analysis of Kyoto Encyclopedia of Genes and Genomes (KEGG) pathways between high- and low-risk groups. (*p-value < 0.05. BP, biological process. CC, cellular component. MF, molecular function).

Figures 4A, B showed the top DEGs between high- and low-risk groups. High expression of IGF2, IGLON5, MEGF8, HSPG2, ALDH1A2, NCCRP1 was associated with high-risk group, while high expression of SLC7A11, PLA2G12A, SST, MMP10, KRT14, PAX2 was associated with low-risk group (Figures 4A, B; Supplementary Table S2). The result of GSEA showed that multiple pathways related with malignant biological behavior of tumors were remarkably activated in high-risk group, including hypoxia, epithelial mesenchymal transition (EMT), TGF-beta signaling, WNT-beta-catenin signaling, and angiogenesis (Figures 4C, D); while pathways associated with immune response, including interferon alpha response, interferon gamma response, and oxidative phosphorylation, were enriched in low-risk group (Figure 4E).

GO enrichment analysis revealed that pathways like integrin binding, connective tissue development, and sulfur compound binding were associated with the disulfidptosis-related risk stratification, which were related to the formation and function of cytoskeleton as well as the disulfide bond (Figure 4F). KEGG enrichment analysis indicated that pathways like PI3K-Akt signaling were enriched in high-risk group, and it is worth noting that these enriched pathways have been demonstrated to be closely related to cancer development (Figure 4G).

Furthermore, correlation analysis of the DRPS showed that most of the genes in the DRPS had a significant correlation between pairwise (Supplementary Figure S1). GO/KEGG enrichment analysis showed that DRPS was mainly associated with the actin related pathways. In addition, the pathways related to sulfur transport were also significantly enriched, indicating that genes included in DRPS might participated in disulfidptosis in OC (Supplementary Figure S1). Sensitivity analysis of the DRPS revealed that good separation could be found between high- and low-risk groups in both the training and validation sets (Supplementary Table S3).

### 3.4 Landscape of tumor infiltrating immune cells and immunotherapy response prediction

**FIGURE 5:**
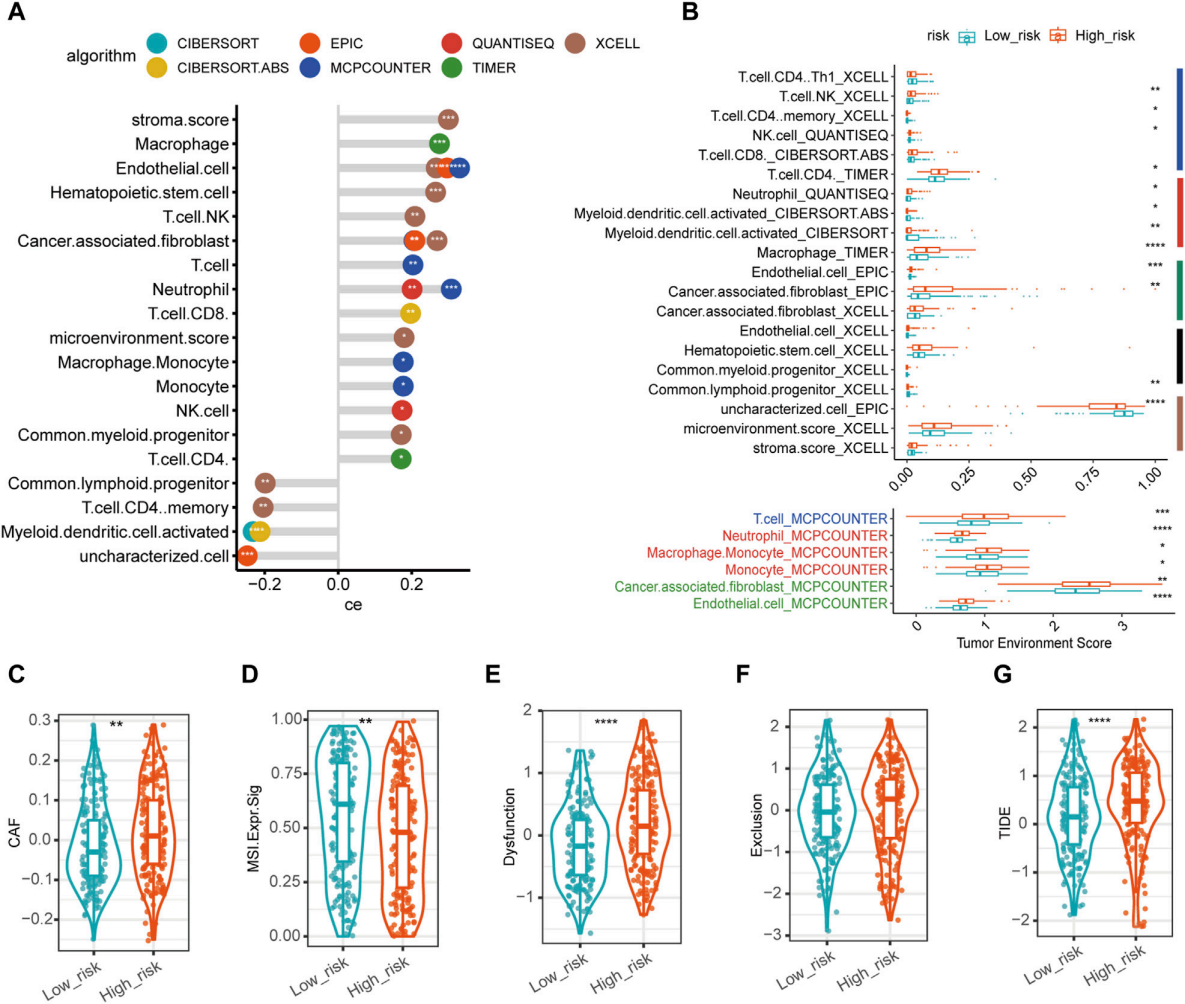
Characteristics of tumor microenvironment in disulfidptosis-related high- and low-risk groups. **(A)** Correlation between microenvironment cell proportion/enrichment score and disulfidptosis-related risk score with top significance. The *x*-axis represented the Spearman correlation coefficient (ce) between the cell type fraction/enrichment score and disulfidptosis-related risk score. The *y*-axis represented different cell proportion/enrichment score in the tumor microenvironment. The different colors of circles represented different algorithms to estimate the immune infiltration in tumor tissues. * in circles represented the statistical significance. The larger the ce was, the more positive correlation was found between the corresponding cell proportion/enrichment score and disulfidptosis-related risk score. **(B)** The infiltration of immune cell proportion/enrichment score between high- and low-risk groups which were top significant between groups. The right colored bars represented major cell types (navy for lymphocytes, red for myeloid cells, green for stroma cells, black for progenitor cells or stem cells, brown for others). **(C–G)** The expression of immunotherapy predictive markers in high- and low-risk groups. (****p-value < 0.0001, ***p-value < 0.001, **p-value < 0.01. *p-value < 0.05. CAF, cancer associated fibroblast. MSI. Expr.Sig, microsatellite instability expression signature. TIDE, tumor immune dysfunction and exclusion score).

Correlation coefficients between the disulfidptosis-related risk score and various immune cells were calculated (Figure 5A). Interestingly, macrophages, neutrophils, and cancer associated fibroblasts (CAFs) were positively correlated with the disulfidptosis-related risk score of patients with OC in more than one algorithm, indicating that high-risk group exhibited more infiltration of these immune cells and these cells promoted the immunosuppressive microenvironment. Conversely, activated myeloid dendritic cells and CD4 memory T cells were negatively correlated with the disulfidptosis-related risk score, indicating that tumors in low-risk group tended to be activated myel infiltrated by these kinds of immune cells. The relative abundances of various immune cells were also estimated between high- and low-risk groups (Figure 5B), which is consistent with the results presented in Figure 5A.

Moreover, we explored biomarkers which have been widely accepted for ICI response prediction. CAF score in high-risk group was significantly higher than in low-risk group (Figure 5C); in contrast, MSI (microsatellite instability) expression signature was significantly lower in high-risk group (Figure 5D). Besides, TIDE score was significantly higher in high-risk group than in low-risk (Figure 5G), in which TIDE dysfunction scores in high-risk group was significantly higher (Figure 5E); whereas TIDE exclusion scores were higher in high-risk group than in low-risk group (Figure 5F) though without statistically significant differences. In total, these biomarkers suggested that high-risk group might have a lower response rate for ICI than low-risk group.

**FIGURE 6:**
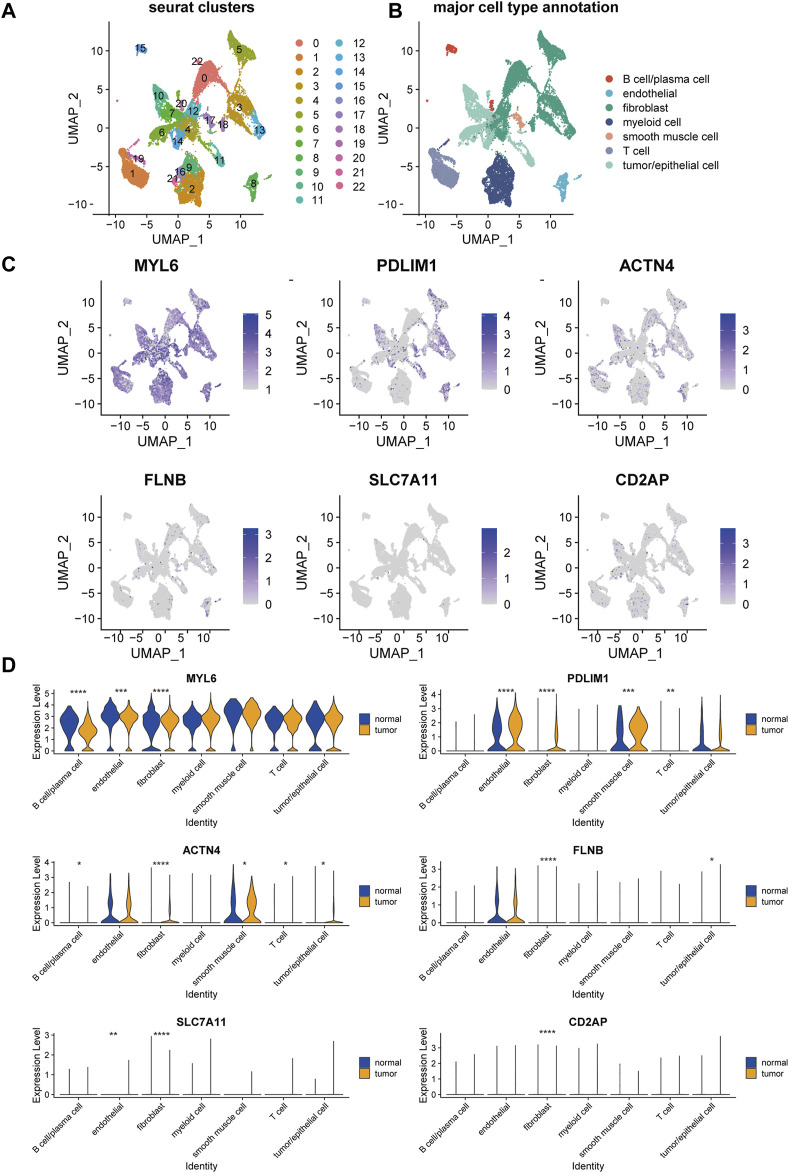
Single-cell landscape of disulfidptosis-related risk signature in epithelial ovarian cancer. UMAP visualization of the clustering of cells from tumor and normal samples from patients with epithelial ovarian cancer, color coded by Seurat cluster **(A)** or major cell type annotation **(B)**. **(C)** Overlay of expression of genes included in disulfidptosis-related risk signature. **(D)** The violin plots of the expression of genes included in disulfidptosis-related risk signature in each major cell type across tumor and normal samples. (UMAP, Uniform Manifold Approximation and Projection. ****p-value < 0.0001, ***p-value < 0.001, **p-value < 0.01, *p-value < 0.05).

To further elucidate the expression of the genes in DRPS in cell level, we mapped these six genes on the UMAP plot of a public single-cell RNA dataset of OC after unsupervised clustering and annotation (Figures 6A, B). The expression bubble plots of marker genes for cell type clustering were displayed in Supplementary Figure S2. As the plot presented, the major types of cells showed distinct marker genes expression signatures (Supplementary Figure S2B), which is consistent with the previous published researches (Dinh et al., 2021; Jia et al., 2023).

MYL6 was commonly expressed in all the seven major types of cells, which had a significantly higher expression level in fibroblasts, endothelial cells, and B cells/plasma cells in tumor compared with normal tissue (Figure 6D). PDLIM1 and ACTN4 were mainly expressed in endothelial cells, fibroblasts, smooth muscle cells, and tumor/epithelial cells (Figure 6C). Besides, these two genes showed markable higher expression in the cell types mentioned about in tumor than normal tissue (Figure 6D). FLNB, SLC7A11, and CD2AP were lowly expressed in almost all major cell types (Figure 6C), but significant expression difference was also found in fibroblasts between tumor and normal tissue.

### 3.5 Drug sensitivity and key genes exploration

**FIGURE 7:**
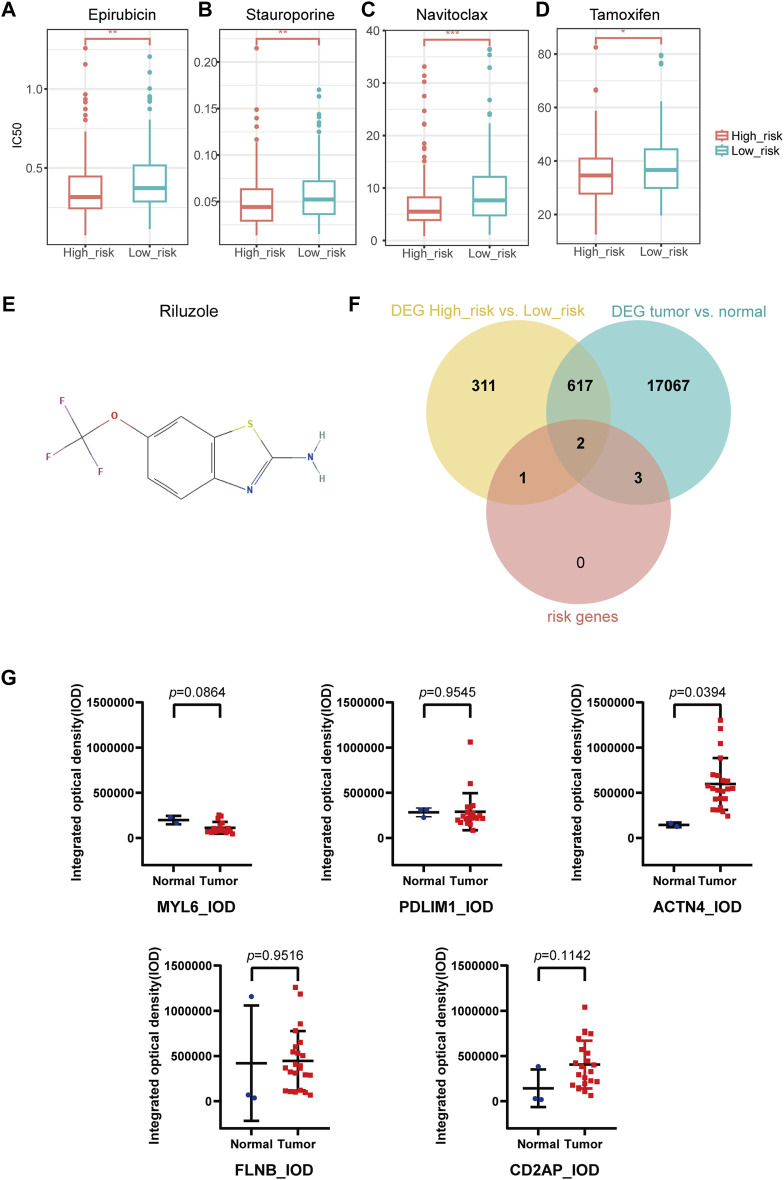
Drug sensitivity prediction, key gene selection and immunohistochemical staining analysis. **(A–D)** IC50 prediction in Epirubicin **(A)**, Stauroporine **(B)**, Navitoclax **(C)**, Tamoxifen **(D)**. **(E)** Potential targeted drug predicted with DGIdb database. **(F)** Two key genes were selected from the intersection of DEGs of high- and low-risk groups, DEGs of tumor and normal groups, and the disulfidptosis-related risk signature (SLC7A11, ACTN4). **(G)** Human Protein Atlas immunohistochemical staining analysis by ImageJ. The integrated optical density of ACTN4 + cells in ovarian cancer and normal ovary was statistically significant. The integrated optical density of MYL6, PDLIM1, FLNB and CD2AP were not statistically significant. (DEGs, differentially expressed genes).

IC50 of 198 drugs in GDSC database in the TCGA-OV cohort was predicted using the “oncoPredict” package (Supplementary Table S4). IC50 of epirubicin, stauroporine, navitoclax, and tamoxifen were significantly lower in high-risk group than in low-risk group (Figures 7A–D), indicating that these drugs might be the potential treatment for high-risk patients. According to the prediction of DGIdb, riluzole (Figure 7E) as the interacted drug of SLC7A11 was the potential targeted drug for patients in high-risk group.

We took the intersection of the DEGs between high- and low-risk groups, DEGs between OC and normal ovary (Supplementary Table S5), as well as the DRPS. Finally, two genes (SLC7A11 and ACTN4) were selected as key genes for further research (Figure 7F; Supplementary Table S6).

### 3.6 Immunohistochemical staining validation by external database

We quantitated IHC staining images from the HPA database using ImageJ software. Since no IHC staining images of SLC7A11 could be found, only other five genes were analyzed for expression at the IHC level. The results revealed significant differences in expression of ACTN4 (*p*-value = 0.0394 for IOD; *p*-value = 0.3597 for AOD) according to Figure 7G and S3. CD2AP (*p*-value = 0.1142 for IOD; *p*-value = 0.4096 for AOD), FLNB (*p*-value = 0.9516 for IOD; *p*-value = 0.2837 for AOD), MYL6 (*p*-value = 0.0864 for IOD; *p*-value = 0.2330 for AOD), PDLIM1 (*p*-value = 0.9545 for IOD; *p*-value = 0.0522 for AOD) showed no significant differences in protein expression between tumor and normal tissues (Figure 7G; Supplementary Figure S3).

## 4 Discussion

OC is the deadliest malignancy in gynecology (Siegel et al., 2023), but the mechanism of its initiation and progression is still not clearly elucidated. The discovery of new prognostic biomarkers and treatments is in the focus of OC research. Disulfidptosis, as a recently discovered novel mode of RCD (Liu et al., 2023), could be the potential regulatory mechanism and therapeutic potential for diseases, which has attracted lots of attention of medical scientists. However, the impact of disulfidptosis on the OC remains unclear. In the present study, we developed a DRPS consisting of six genes for OC and stratified patients with OC into the high- and low-risk groups according to the disulfidptosis-related risk score to predict the survival. Our study also revealed that the DRPS could be linked to initiation and metastasis of OC, correlate with specific patterns of immune cell infiltration, and indicate potential drugs for patients with different risk stratifications.

The DRPS included six genes (MYL6, PDLIM1, ACTN4, FLNB, SLC7A11, and CD2AP). SLC7A11, as a key disulfidptosis regulator, participates in the uptake of extracellular cystine and the biosynthesis of glutathione to protect the cells from the damage caused by oxidative stress (Koppula et al., 2018). However, the exact role of SLC7A11 in OC remained controversial. Yang et al. (2022) showed that OC patients with high SLC7A11 expression have a favorable prognosis, while Wu et al. (2022) found that SLC7A11 was a risk factor for OS and progression-free survival (PFS) in OC. In this research, we found SLC7A11 was an independent prognostic protective factor for OC according to the multivariate cox regression analysis of OS, which provided new evidence on this controversial issue. Under conditions of glucose deficiency, SLC7A11^high^ cells exhibited a decrease in NADPH production, resulting in the accumulation of cystine and subsequent formation of abnormal disulfide bonds. These aberrant bonds then targeted the actin cytoskeleton, leading to its collapse, which eventually resulted in disulfidptosis (Liu et al., 2023). Cancer cells, which are programmed to consume large amounts of glucose, frequently lead to glucose depletion in the tumor microenvironment, therefore the low expression of SLC7A11 in cancer cells could inhibit disulfidptosis, then promote tumor growth and progression, and further contribute to the poor prognosis of patients with cancer (Figures 2C, 6D) (Liu et al., 2021). SLC7A11 is also a star molecule in ferroptosis, its overexpression promotes ferroptosis resistance (Dixon et al., 2012; Jiang et al., 2015), and studies on the relationship between SLC7A11 and TME mainly focus on ferroptosis-related pathways (Cheng et al., 2022; Tang et al., 2023; Zhao et al., 2023). Whether SLC7A11 could independently influence tumor progression and TME modulation by disulfidptosis needs to be further investigated.

The other five genes included in the signature belong to actin-related proteins, which modulate the assembly of conventional actin and contribute to microtubule-based motility (Schafer and Schroer, 1999). Among them, FLNB and CD2AP were significantly associated with the prognosis of OC patients. The role of FLNB in cancer development is still a mysterious challenge. A previous study suggested that knockdown of FLNB in cancer cells decreased di-phosphorylation of MRLC and phosphorylation of FAK, then inhibited cell migration and focal adhesions (Iguchi et al., 2015). Besides, our results suggested FLNB was mainly high-expressed in endothelial cell. It has been reported that inhibition of FLNB could reduce endothelial cell migration and VEGF-induced cell migration, which could inhibit angiogenesis *in vitro* and reduce tumor development (Del Valle-Perez et al., 2010). However, zebrafish and mouse model systems showed that silencing FLNB increased MMP-9 expression in endothelial and cancer cells, which enhanced tumor angiogenesis and VEGF-A secretion, then promoted tumor growth and metastasis (Bandaru et al., 2014). In this study, we found that FLNB was an independent prognostic risk factor for OC, aligned with previous research in lung adenocarcinoma (Ni et al., 2023). Still, the mechanism of FLNB in disulfidptosis in cancer deserves further investigation. CD2AP, another independent prognostic factor, was regarded as a scaffolding molecule which regulates cytoskeletal molecules and signal transduction (Lehrer and Rheinstein, 2020). CD2AP has been reported to inhibit tumor metastasis by promoting cell adhesion and cytoskeleton assembly in gastric cancer (Xie et al., 2020). In the present study, CD2AP was positively correlated with longer OS (Figures 2C, 6C), which was consistent with the effect in gastric cancer (Xie et al., 2020). A study found that inactivation of CD2AP promoted the differentiation of CD4 T cells toward the follicular helper lineage in chronic lymphocytic choriomeningitis virus infection (Raju et al., 2018). Taken together, CD2AP could modulate both cancer cells and immune cells. ACTN4, which was one of the key genes found by this study (Figure 7F), was known to affect cell cycle, cell motility, and regulation of nuclear transcription factor activity (Tentler et al., 2019). Previous studies proved that high expression of ACTN4 not only promoted cell motility and invasion, but was also correlated with poor prognosis and chemoresistance of various tumors including OC (Yamamoto et al., 2007; Barbolina et al., 2008; Kikuchi et al., 2008; Yamamoto et al., 2009; Yamada et al., 2010; Wang et al., 2015), which supported our finding that ACTN4 was associated with poor prognosis. After adjusting for confounders, MYL6 and PDLIM1 tended to be protective factors for OS in OC, and the expression of them were both upregulated in OC tissues. The role of MYL6 in OC is currently unknown, but it has been shown that MYL6 expression was upregulated in rhabdomyosarcoma (Eichenmüller et al., 2007), and was negatively associated with cell migration in melanoma (Vierthaler et al., 2022). Previous studies suggested that PDLIM1 was upregulated in OC (Qiu et al., 2021), which was consistent with our findings (Figure 6D). PDLIM1 could inhibit tumor metastasis and EMT by interacting with E-cadherin/β-catenin adhesion complex, inhibiting the Hippo signaling pathway (Chen et al., 2016; Huang et al., 2020). How genes in DRPS influence the survival of OC requires further experimental confirmation.

Currently, there is an emerging trend to develop biomarker for prognostic prediction or molecular subtyping to guide the precise management for OC. Such as the Cancer Genome Atlas Research Network proposed a classic four-classification subtypes of OC based on transcriptome in 2011, which described the gene expression content but did not differ in survival or treatment options (Cancer Genome Atlas Research, 2011). With the rapid development of oncology, molecular subtyping and prognostic models based on new mechanisms of tumor development, such as EMT, TME, or ferroptosis, have gradually been proposed. Here, we sought to construct a new prognostic signature and risk stratification system of OC based on disulfidptosis, which might provide assistance in clinical prognostic assessment, mining immune infiltration characteristics, and finding potential targeted drugs. This signature was proven to be an independent predictor of OS after adjustment for clinicopathological factors (Figure 2H), and the corresponding nomogram achieved good performance in training and validation sets (Figures 2I–K), indicating the clinical practical value of this nomogram. High-risk group had a relatively lower disulfidptosis level (Figure 3I). Besides, the risk stratification did not seem to correlate with gene mutations or clinical-pathological factors (Figures 3A–H), and the difference between high- and low-risk groups might arise from transcriptomic level (Figures 4C–G). It is worth noting that sulfur compound binding was differentially enriched between two groups (Figure 4F). As we mentioned earlier, intracellular disulfide stress would induce disulfidptosis of cells. The difference in the pathway of sulfur compound binding might indicate the difference in intracellular disulfide stress, which echoed the mechanism of disulfidptosis.

TME also showed different characteristics in different risk groups. Increased infiltration of neutrophils and CAFs were observed in high-risk group, whereas activated myeloid dendritic cells and CD4 memory T cells had higher abundance in low-risk group. Preclinical studies have revealed that CAFs can promote angiogenesis and tumor progression in many tumors including OC (O’Connell et al., 2011; Bhowmick et al., 2004; Zhang et al., 2011). The more CAFs were infiltrated in TME, the stronger their impact in promoting tumor progression, leading to a worse prognosis. It was confirmed that neutrophils were associated with poor OS and PFS of patients with OC (Yang et al., 2020), and this correlation had also been demonstrated in melanoma and hepatocellular carcinoma (Li et al., 2011; Jensen et al., 2012). While dendritic cells and CD4 memory T cells had also found to be associated with anti-tumor immunity in TME and often suggested a better prognosis (Chen et al., 2020; Liu J. et al., 2021; Duong et al., 2022), which was also consistent with our findings.

We also found that high-risk group might have a lower response rate for ICI compared with low-risk group by multiple biomarkers. High-risk group may be more sensitive to epirubicin, stauroporine, navitoclax, and tamoxifen with lower IC50 (Figures 7A–D). These findings suggested that ICI might not be a preferable choice for OC patients in high-risk group, but the above-mentioned chemotherapy drugs might deserve consideration. In addition, riluzole might be a potential targeted drug for high-risk group. Riluzole is a glutamate release inhibitor, known as a neuroprotective, anticonvulsant, and sedative drug, that has been approved for the treatment of amyotrophic lateral sclerosis since 1995 (Doble, 1996). Studies have shown that riluzole inhibits multiple malignancies including melanoma, pancreatic cancer, breast cancer, and cisplatin-resistant lung cancer cells in a dose-dependent manner by inducing cell cycle arrest and so on (Speyer et al., 2016; Wangpaichitr et al., 2017; Shin et al., 2018; Sun et al., 2021). However, the role of riluzole in OC has not been evaluated so far. Whether this drug can affect the development of OC through disulfidptosis needs to be clarified in our further study.

This study based on multi-omics data revealed the relationship between the initiation and progression of OC and disufidptosis. One limitation of this study was that this study mainly focused on the transcriptive level analysis, and the exploration on the genomic level of DRPS was relatively not deep enough. GWAS-based analysis like Mendelian randomization merits further exploration to further reveal the correlations between OC development and genetic variants of the genes in DRPS. Another limitation was we didn’t verify the results with cell or animal experiments limited by the experimental condition and specimen. Still, we believe this study might be a necessary starting point for research in this direction. DRPS could predict OS and immunotherapy efficacy for clinical application; enrichment analysis and immune cell infiltration analysis could be explored for further researches on disease mechanisms. In addition, drug sensitivity analysis found some drugs for high-risk patients, indicating potential clinical value for individualized treatment. Carefully designed *in vitro* cell studies and animal experiments, as well as scientifically rigorous clinical trials will be needed to validate these findings.

In the present study, we developed a DRPS and corresponding prognostic nomogram for OC, which was important for prognostic assessment, TME modification, drug sensitivity prediction, and exploration of potential mechanisms in tumor development.

## Data Availability

The original contributions presented in the study are included in the article/Supplementary Material, codes for analysis and data are available online at https://github.com/GuangyaoCai/disulfidptosis_oc/.
