# Supplementary material for: Disulfidptosis-related signature elucidates the prognostic, immunologic, and therapeutic characteristics in ovarian cancer
Source: Front Genet. 2024 Apr 17;15:1378907. doi: 10.3389/fgene.2024.1378907 (PMC11061395; doi:10.3389/fgene.2024.1378907)
Supplement: Supplementary file 1 [file DataSheet1.ZIP › Supplementary materials/Supplementary_Materials.docx]

Supplementary Materials

## Supplementary Figures


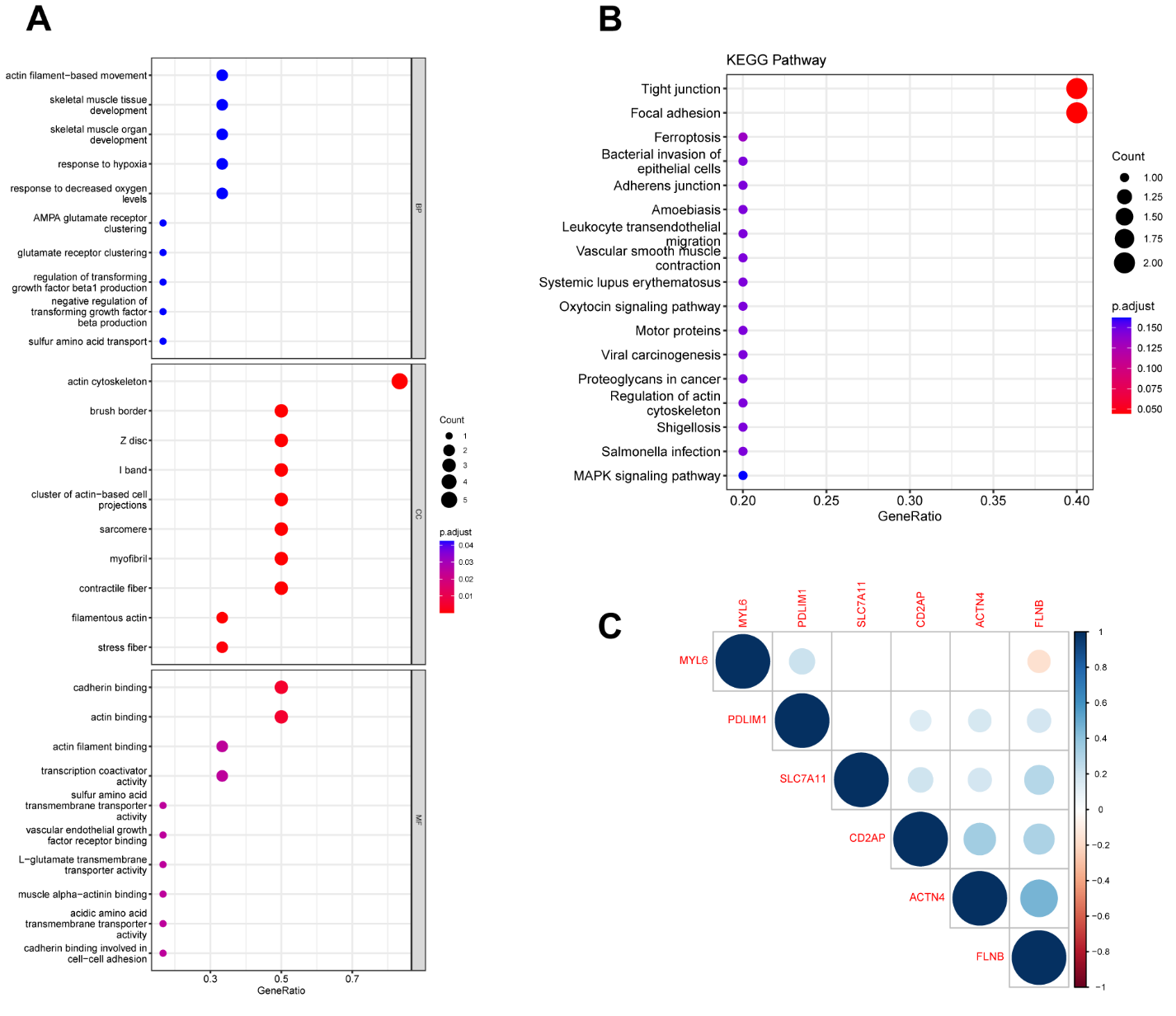


**FIGURE S1**

GO/KEGG enrichment analysis and correlation analysis of DRPS. **(A)** Enrichment analysis of GO pathways of DRPS in OC. **(B)** Enrichment analysis of KEGG pathways of DRPS in OC. **(C)** The correlation analysis of DRPS. The color and the size of circles represented the Pearson correlation coefficients between genes in DRPS. Blue represents positive correlation between the two features. The bluer the color is, the higher positive correlation coefficient is. Red represents negative correlation. The redder the color is, the higher negative correlation coefficient is. Only coefficients with a *p* < 0.05 were presented on the plot. Coefficients with a *p* value ≥ 0.05 were blank on the plot. (DRPS, disulfidptosis-related prognostic signature. OC, ovarian cancer. GO, Gene Ontology. KEGG, Kyoto Encyclopedia of Genes and Genomes.)


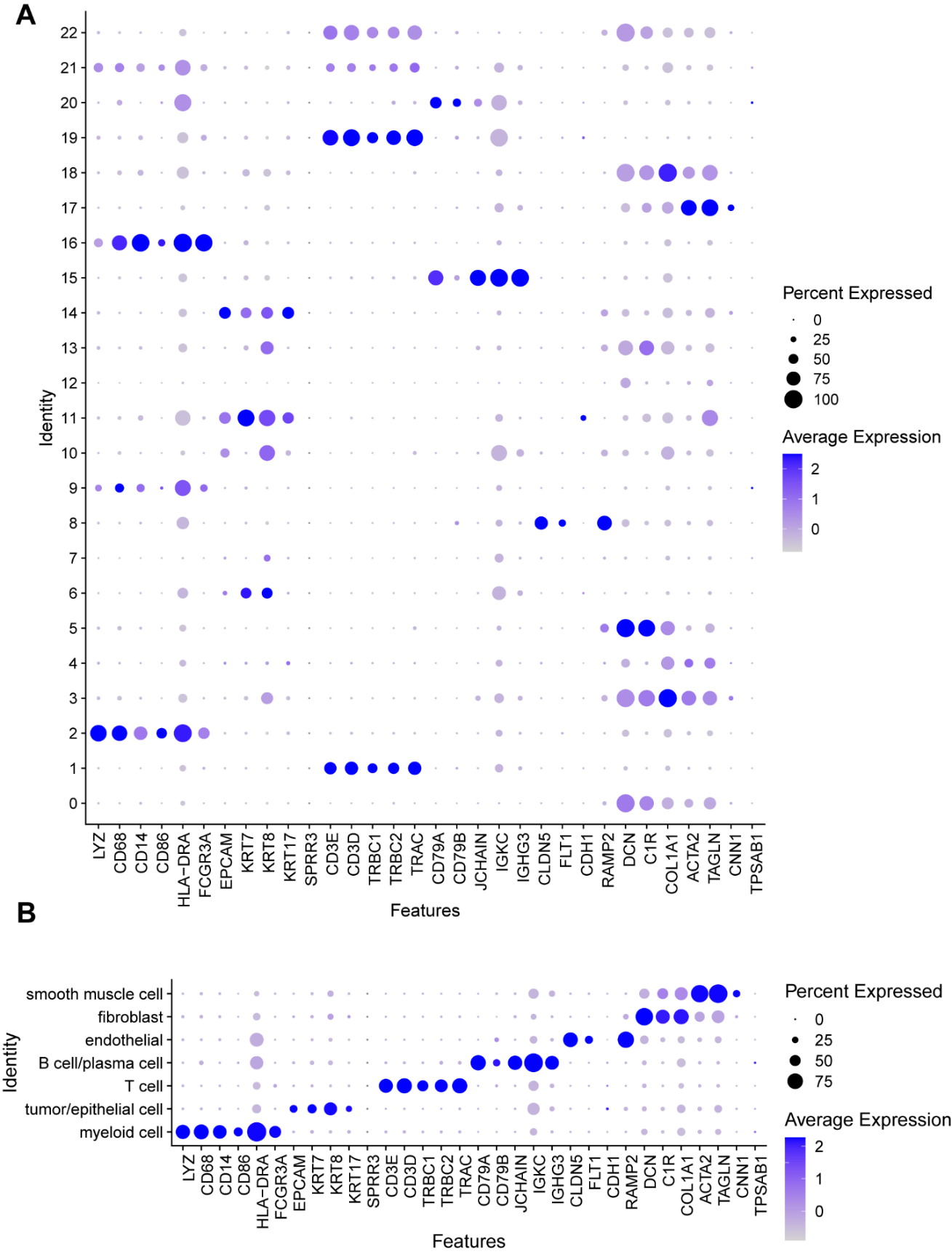


**FIGURE S2**

The expression bubble plots of marker genes for single-cell annotation clustering. **(A)** Bubble plot of marker gene expression in 23 clusters. **(B)** Bubble plot of marker gene expression in the annotated major cell types.


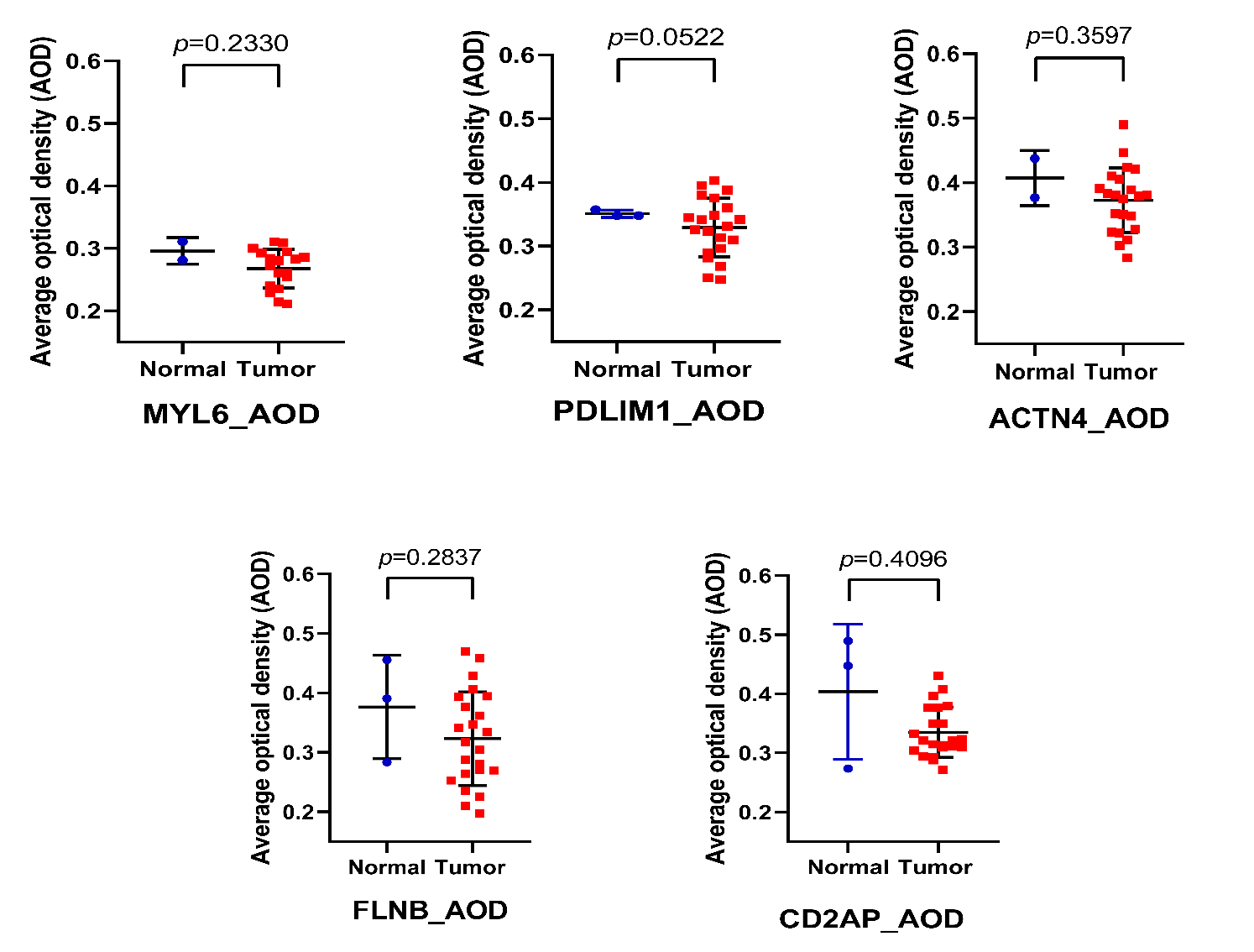


**FIGURE S3**

Human Protein Atlas immunohistochemical staining analysis by Image J. The average optical density (AOD) of ACTN4, MYL6, PDLIM1, FLNB and CD2AP+ were not statistically significant.

## Supplementary Tables

**Table S1** Baseline characteristics of the included cohorts.

**Table S2** Differentially expressed genes (DEGs) between high- and low-risk groups.

**Table S3** Sensitivity analysis of DRPS using copula-based methods.

**Table S4** Drugs with significant IC50 difference in GDSC database in the TCGA-OV cohort.

**Table S5** Differentially expressed genes (DEGs) between ovarian cancer (OC) and normal ovary.

**Table S6** The intersection of the differentially expressed genes (DEGs) between high- and low-risk groups, DEGs between OC and normal ovary, as well as the disulfidptosis-related prognostic signature (DRPS).

## Other Materials

**Supplementary** R script.
