# Supplementary material for: Disulfidptosis-related signature elucidates the prognostic, immunologic, and therapeutic characteristics in ovarian cancer
Source: Front Genet. 2024 Apr 17;15:1378907. doi: 10.3389/fgene.2024.1378907 (PMC11061395; doi:10.3389/fgene.2024.1378907)
Supplement: Supplementary file 1 [file DataSheet1.ZIP › Supplementary materials/Table S1.docx]

| **Table S1** Baseline characteristics of the included cohorts | | | |
| --- | --- | --- | --- |
|  | **TCGA-OV** | **GSE9891** | **E-MTAB-386** |
| **N** | 374 | 285 | 129 |
| **Age (years)** | 59(51-68) | 59(53-68) | NA |
| **FIGO stage** |  |  |  |
| Stage I | 1 (0.3%) | 24 (8.4%) | 0 (0%) |
| Stage II | 22 (5.9%) | 18 (6.3%) | 1 (0.8%) |
| Stage III | 291 (77.8%) | 217 (76.1%) | 109 (84.5%) |
| Stage IV | 57 (15.2%) | 22 (7.7%) | 19 (14.7%) |
| NA | 3 (0.8%) | 4 (1.4%) | 0 (0%) |
| **Histological subtypes** |  |  |  |
| Epithelial | 374 (100%) | 285 (100%) | 129(100%) |
| Serous | 374 (100%) | 264 (92.6%) | 129(100%) |
| Mucinous | 0 (0%) | 0 (0%) | 0 (0%) |
| Endometrioid | 0 (0%) | 20 (7.0%) | 0 (0%) |
| Clear cell carcinoma | 0 (0%) | 0 (0%) | 0 (0%) |
| Other | 0 (0%) | 1 (0.4%) | 0 (0%) |
| Non-epithelial | 0 (0%) | 0 (0%) | 0 (0%) |
| NA | 0 (0%) | 0 (0%) | 0 (0%) |
| **Grade** |  |  |  |
| 1 | 0 (0%) | 19 (6.7%) | 0 (0%) |
| 2 | 42 (11.2%) | 97 (34.0%) | 0 (0%) |
| 3 | 316 (84.5%) | 164 (57.5%) | 129 (100%) |
| NA | 16 (4.3%) | 5 (1.8%) | 0 (0%) |
| **Residual disease** |  |  |  |
| Optimal | 231 (61.7%) | 160 (56.2%) | 98(76.0%) |
| R0 | 63 (16.8%) | 84 (29.5%) | NA |
| R1 | 168 (44.9%) | 76 (26.7%) | NA |
| Not optimal | 95 (25.4%) | 70 (24.6%) | 28(21.7%) |
| NA | 48 (12.8%) | 55 (19.3%) | 3(2.3%) |

Median (IQR) and frequencies (%) were assessed for continuous and categorical variables, respectively. (NA, not available. R0, complete cytoreduction. R1, residual disease of ≤ 1 cm. IQR, interquartile range.)
