# Supplementary material for: Disulfidptosis-related signature elucidates the prognostic, immunologic, and therapeutic characteristics in ovarian cancer
Source: Front Genet. 2024 Apr 17;15:1378907. doi: 10.3389/fgene.2024.1378907 (PMC11061395; doi:10.3389/fgene.2024.1378907)
Supplement: Supplementary file 1 [file DataSheet1.ZIP › Supplementary materials/Table S3.docx]

|  |  | **TCGA-OV** | | |  | **GSE9891** | | |  | **E-MTAB-386** | | |
| --- | --- | --- | --- | --- | --- | --- | --- | --- | --- | --- | --- | --- |
|  |  | **Median survival time of high-risk group** | **Median survival time of low-risk group** | ***p* value** |  | **Median survival time of high-risk group** | **Median survival time of low-risk group** | ***p* value** |  | **Median survival time of high-risk group** | **Median survival time of low-risk group** | ***p* value** |
| **Clayton copula** | α=0 (τ=0) | 46.09 | 68.68 | <0.0001 |  | 53.74 | 77.84 | 0.017 |  | 36.58 | 46.43 | 0.033 |
|  | α=5 (τ=0.71) | 36.14 | 51.52 | <0.0001 |  | 34.75 | 38.26 | 0.283 |  | 31.80 | 38.81 | 0.017 |
|  | α=10 (τ=0.83) | 35.16 | 49.88 | <0.0001 |  | 33.63 | 36.48 | 0.350 |  | 31.27 | 37.74 | 0.017 |
|  | α=15 (τ=0.88) | 34.75 | 49.22 | <0.0001 |  | 33.26 | 35.78 | 0.367 |  | 31.10 | 37.34 | 0.017 |
| **Gumbel copula** | α=1 (τ=0.5) | 39.02 | 57.08 | <0.0001 |  | 41.33 | 51.04 | 0.100 |  | 34.24 | 41.22 | 0.067 |
|  | α=6 (τ=0.86) | 35.03 | 50.1 | <0.0001 |  | 34.29 | 37.31 | 0.317 |  | 31.67 | 37.62 | 0.067 |
|  | α=11 (τ=0.92) | 34.46 | 49.03 | <0.0001 |  | 33.36 | 35.71 | 0.433 |  | 31.23 | 37.09 | 0.033 |
|  | α=16 (τ=0.94) | 34.26 | 48.62 | <0.0001 |  | 33.03 | 35.15 | 0.450 |  | 31.07 | 36.89 | 0.033 |
| **Frank copula** | α=2 (τ=0.21) | 42.78 | 63.12 | <0.0001 |  | 47.87 | 65.02 | 0.017 |  | 35.50 | 44.18 | 0.050 |
|  | α=7 (τ=0.56) | 38.56 | 56.01 | <0.0001 |  | 40.39 | 49.63 | 0.067 |  | 33.57 | 40.49 | 0.067 |
|  | α=12 (τ=0.71) | 37.01 | 53.32 | <0.0001 |  | 37.45 | 44.10 | 0.100 |  | 32.60 | 38.97 | 0.067 |
|  | α=17 (τ=0.79) | 36.24 | 51.9 | <0.0001 |  | 35.98 | 41.36 | 0.117 |  | 32.08 | 38.25 | 0.067 |
| Reference: Yeh C-T, Liao G-Y, Emura T. Sensitivity Analysis for Survival Prognostic Prediction with Gene Selection: A Copula Method for Dependent Censoring. Biomedicines 2023, 11(3): 797. (DRPS, disulfidptosis-related prognostic signature.) | | | | | | | | | | | |  |

**Table S3** Sensitivity analysis of DRPS using copula-based methods
